# Autosomal dominant pseudohypoaldosteronism type 1 with a novel splice site mutation in *MR *gene

**DOI:** 10.1186/1471-2369-10-37

**Published:** 2009-11-14

**Authors:** Kyoko Kanda, Kandai Nozu, Naoki Yokoyama, Ichiro Morioka, Akihiro Miwa, Yuya Hashimura, Hiroshi Kaito, Kazumoto Iijima, Masafumi Matsuo

**Affiliations:** 1 Department of Pediatrics, Kobe University Graduate School of Medicine, Kobe, 6500017, Hyogo, Japan

## Abstract

**Background:**

Autosomal dominant pseudohypoaldosteronism type 1 (PHA1) is a rare inherited condition that is characterized by renal resistance to aldosterone as well as salt wasting, hyperkalemia, and metabolic acidosis. Renal PHA1 is caused by mutations of the human mineralcorticoid receptor gene (*MR*), but it is a matter of debate whether *MR *mutations cause mineralcorticoid resistance via haploinsufficiency or dominant negative mechanism. It was previously reported that in a case with nonsense mutation the mutant mRNA was absent in lymphocytes because of nonsense mediated mRNA decay (NMD) and therefore postulated that haploinsufficiency alone can give rise to the PHA1 phenotype in patients with truncated mutations.

**Methods and Results:**

We conducted genomic DNA analysis and mRNA analysis for familial PHA1 patients extracted from lymphocytes and urinary sediments and could detect one novel splice site mutation which leads to exon skipping and frame shift result in premature termination at the transcript level. The mRNA analysis showed evidence of wild type and exon-skipped RT-PCR products.

**Conclusion:**

mRNA analysis have been rarely conducted for PHA1 because kidney tissues are unavailable for this disease. However, we conducted RT-PCR analysis using mRNA extracted from urinary sediments. We could demonstrate that NMD does not fully function in kidney cells and that haploinsufficiency due to NMD with premature termination is not sufficient to give rise to the PHA1 phenotype at least in this mutation of our patient. Additional studies including mRNA analysis will be needed to identify the exact mechanism of the phenotype of PHA.

## Background

Pseudohypoaldosteronism type 1 (PHA1) is a rare inherited condition that is characterized by renal insensitivity to the action of mineralcorticoids (aldosterone) [1]. At least two forms of PHA1, autosomal dominant and recessive of the disease have been described. However, patients with autosomal dominant PHA1 generally have much milder symptoms of having renal PHA than those with autosomal recessive PHA1 which shows multiple target organ unresponsiveness to mineralcorticoids such as sweat and salivary glands, colon and kidney [2]. Aldosterone plays a crucial role in the regulation of blood pressure and potassium homeostasis. It binds to the mineralcorticoid receptor (MR) in the distal nephron which triggers increased sodium reabsorption via the epithelial sodium channel (ENaC) to restore intravascular volume. Recently, it became clear that mutations in the MR gene (*MR or NR3C2*) leads to autosomal dominant renal PHA1 and mutations in the ENaC alpha, beta and gamma subunit genes (*SCNN1A*, *SCNN1B *and *SCNN1G*) leads to autosomal recessive severe systemic PHA1 [3,4].

Patients with autosomal dominant renal PHA1 generally require oral salt supplementation, but typically show a gradual clinical improvement in renal salt loss during childhood. Some individuals are clinically asymptomatic but may have elevated PRA and aldosterone levels [2]. In most cases, renal PHA1 is the result of mutations of the gene *MR*, which consists of 10 exons [5]. The *MR *gene contains an amino-terminal region that harbors a ligand-independent transactivation function coded by exon 2 and a conserved DNA binding domain encoded by exon 3-4 and a ligand binding domain responsible for ligand binding and ligand-dependent transactivation encoded by exons 5-9 [6]. To date more than 50 different PHA1-causing mutations in the *MR *gene have been identified, including nonsense, frameshift, missense, and splice site mutations distributed throughout the gene [7-15].

This report concerns a patient with PHA1 with a novel splice acceptor site mutation which leads to exon skipping and frame shift result in premature termination at the transcript level. The mRNA from peripheral blood lymphocytes and urinary sediments showed evidence of wild type and exon-skipped RT-PCR products.

## Methods

### Case report

The female index patient was born prematurely after 34 weeks of gestation by cesarean section necessitated by premature membrane rupture. At birth she weighed 1965 g and presented with respiratory distress because of transient tachypnea of the newborn. Initial laboratory examinations showed hyponatremia (127 mmol/L), hyperkalemia (6.5 mmol/L), and extremely elevated plasma rennin activity (PRA) (>20 ng/ml/hr; normal: <5.4 ng/ml/hr), and plasma aldosterone concentration (16,000.0 pg/ml; normal: <240 pg/ml) were noted. 17-OHprogesterone, adrenocorticotropic hormone (ACTH) and cortisol levels were normal. After her respiratory distress had improved, the patient showed failure to thrive and a persistently low serum sodium level. The clinical and laboratory findings led us to suspect PHA1. At 13 days of age, a very low salt supplement (0.5 g/day) was started, after which the electrolyte disturbance was resolved accompanied by gradual clinical improvement with a normal growth rate so that sodium supplementation could be discontinued at 7 months. The patient is currently 8 months old and in good condition, but aldosterone levels have remained elevated. Her parents were without any clinical symptoms. Her mother showed normal plasma electrolytes concentration, but elevated PRA (7.5 ng/ml/hr) and aldosterone level (931 pg/ml) were noted.

This study was approved by the Institutional Review Board of Kobe University Graduate School of Medicine and written informed consent for this study was obtained from the patient's parents.

### Genomic DNA analysis

Genomic DNA was isolated from peripheral blood leukocytes of the patient and her parents as well as of normal control subjects with the Qiagen kit (Qiagen Inc., Chatsworth, CA, USA), according to the manufacturer's instructions. Eleven pairs of oligonucleotide primers were generated to amplify all eight coding exons of the *MR *gene. Polymerase chain reaction (PCR) was performed and the PCR products were purified with a DNA purification kit (Qiagen Japan, Tokyo, Japan), and then analyzed, including every exon and intron-exon boundary, by direct sequencing with a DNA sequencer (Perkin-Elmer-ABI, Foster City, CA, USA).

### RNA expression analysis

Total RNA was extracted from peripheral blood leukocytes of the patient and her mother and from urine sediments of the mother. The urine sediments were obtained by centrifugation at 1500 rpm for 10 min from 100 mL of early morning urine. Microscopic examination of these sediments confirmed that they contained sufficient renal tubular epithelial cells.

RNA was isolated with the aid of Isogen Kit (Nippon Gene Co., Toyama, Japan), and was then reverse transcribed onto cDNA by using random hexamers and the Superscript III kit (Invitrogen). cDNA was amplified by means of nested PCR with forward primers located in exon 5 or 6, and the reverse primer complementary to exon 8 or 9 (exon 5, forward: TTGCCTCTTGGGACCAAAT; exon 6, forward: TCCTTGAAAACATTGAACCTGA; exon 8, reverse: CCCAGAATTGTTGGGACACT; exon 9 reverse: ATTCCAGCAGGTCGCTCAC). After 35 cycles of amplification, PCR products were separated on 2% agarose and sequenced with a DNA sequencer (Perkin-Elmer-ABI, Foster City, CA, USA). Normal control kidney cDNA was obtained from the Human Kidney cDNA Library (Invitrogen).

## Results

**Figure 1:**
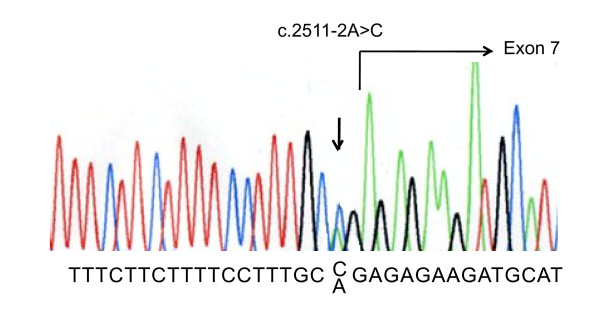
**Nucleotide change**. The nucleotide change identified in the pseudohypoaldosteronism type 1 (PHA1) patient by direct sequencing analysis. A heterozygous transition (a>c) at position -2 bp of the acceptor splice site of intron 6 led to the hypothesis that exon 7 might have been skipped in the transcript.

**Figure 2:**
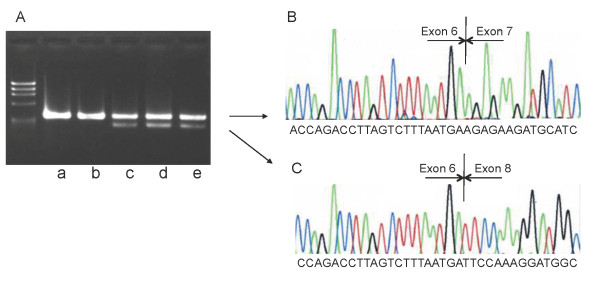
**Electrophoresis of cDNA and a fragment of the sequence cDNA from the patient**. (A) PCR using the forward primer located in exon 6 and the reverse one located in exon 8. Control samples (a: extracted from leukocytes; b: extracted from kidney library) clearly show a single band. The sample from the patient (c: extracted from leukocytes), and her mother (d: extracted from leukocytes; e: extracted from urine sediment) shows two bands, one the same size as the control sample (a and b), and the other smaller. (B) Normal product (large band) shows a normal sequence. (C) The smaller product (small band) shows exon 6 immediately followed by exon 8, so that exon 7 has been skipped. a: control, leukocytes; b: control, kidney library; c: patient, leukocytes; d: mother, leukocytes; e: mother, urine sediment.

Direct sequencing of the PCR-amplified products disclosed one abnormality, namely double peaks of a and c, signaling a heterozygous a to c substitution at position -2 bp of the acceptor splice site of intron 6, c.2511-2A>C (Figure 1). This mutation was also found in her mother. It was therefore hypothesized that exon 7 might have been skipped in the transcript. To confirm the inactivation of the splice-acceptor site, mRNA extracted from peripheral blood leukocytes and urine samples were used to amplify a fragment of exons 6 to 8 by means of RT-PCR using a pair of primers recognizing exon 6 and 8. PCR products from the patient's and her mother's blood samples showed two bands, one the same size as the control and the other shorter (Figure 2a). Sequencing of the larger product showed a normal sequence, but sequencing of the shorter product indicated complete absence of the exon 7 sequences, that is, exon 6 was directly joined to exon 8 (Figure 2b). Exon 7 of the *MR *gene contains 131 bp so that skipping of this exon creates an out-of-frame mutation in mRNA, which in turn introduces a premature stop codon in exon 8. The PCR products from the mother's urine sediments also showed two bands, one the same size as the control and the other shorter, and sequencing yielded the same result as for the patient's and her mother's blood samples.

## Discussion

In this study we identified a novel mutation c.2511-2A>C in intron 6 in the *MR *gene and this mutation was strongly suspected of eliminating a consensus splice acceptor site, resulting in exon 7 skipping in the mRNA. Although no clear evidence has been shown, it is believed that this mutation in the *MR *gene reduces the ligand-binding capacity of *MR *[6]. This same mutation was found in the patient's mother.

Both mother and daughter had a very mild phenotype in comparison with typical patients with PHA1. For this reason, our patient needed only a small amount of sodium replacement (0.5 g/day) and this supplement could be terminated early, at the age of 7 months. Her mother was clinically free of symptoms, had normal plasma electrolytes, and showed only elevated PRA and aldosterone level. No occasion of salt loss, not even during infancy or childhood, was recorded, and she had never undergone treatment as an inpatient. Viemann at al reported that the patients with typical PHA1 need a much larger amount of salt replacement than did our patient, 4 g/day to 12 g/day for therapy [7], and it is reported the patients needed the supplement for a relatively longer time, 1 to 3 years [16]. However, the finding that some patients carrying the *MR *mutation have no symptomatic or very mild symptomatic manifestations can be found in some reports of PHA1 [7,17]. Moreover, distinct differences of the disease severity between various affected members within one family or between unrelated individuals carrying the same mutation could be observed [7,14]. We should consider that salt intake in the diet may modulate the phenotype and that some patients may compensate their phenotype without treatment just by a high salt diet. However, there is another possibility that the phenotype of PHA1 may not originate from the genotype of only the *MR *mutation. As postulated by Geller [5] and Tajima [8], it is possible that either mutations in additional genes or non-genetic factors may contribute to the clinical and biochemical phenotypes of the carriers of *MR *mutations. However, several attempts to pin down these additional genes or factors have been without success [17,18].

About genotype-phenotype correlations, Riepe describes in his recent review article that no association could be drawn from the available clinical data and *MR *gene mutations [19]. This fact may also suggest the existence of other factors.

Many human diseases that are caused by loss-of-function mutations in nuclear receptors have been reported, but the mechanism of the disease differs for each receptor. Moreover, whether *MR *mutations cause mineralcorticoid resistance via haploinsufficiency or dominant negative mechanism remains controversial. To test the pathogenic mechanism, Sartorato et al showed in co-transfection experiments that the two mutated receptors they identified, Q776R and L979P, exerted a dominant negative effect on the wild type receptors, and stated that this dominant negative effect is necessary to give rise to autosomal dominant PHA1 [7]. However, Riepe et al and Balsamo et al performed the same co-transfection experiments and showed that the mutated receptors they identified, C818L, A1012X, Y134X and L772X, exhibited no dominant negative effect on the wild-type *MR *proteins. Therefore, Riepe et al and Balsamo et al claimed that haploinsufficiency of the *MR *locus is sufficient to generate PHA1 in their patients [16,17].

Geller et al had access to RNA analysis to test for nonsense-mediated mRNA decay (NMD) in a patient with a nonsense mutation. They found wild type but not mutant *MR *mRNA in peripheral blood lymphocytes of a PHA1 patient with a nonsense mutation in exon 3 (A590X). The absence of a mutant RNA was probably due to NMD, indicating the patient possessed the onset mechanism of haploinsufficiency because there was no mutant mRNA due to NMD [20].

With genetic analysis using RT-PCR, the problem is sometimes encountered that NMD interferes with the detection of nonsense mutations. The NMD pathway is an mRNA surveillance system that typically degrades transcripts containing premature termination codons (PTCs) to prevent translation of unnecessary or aberrant transcripts. Failure to eliminate these mRNAs with PTCs may result in the synthesis of abnormal proteins that can be toxic to cells through dominant negative or gain-of-function effects. Geller et al suggested that haploinsufficiency is sufficient to generate the PHA1 phenotype because of this mechanism [14]. However, we confirmed by means of RT-PCR that transcripts with exon 7 skipped remain in mRNA from not only peripheral blood lymphocytes but also urinary sediments. Exon 7 of the *MR *gene contains 131 bp and this exon skipping creates an out-of-frame mutation and leads to PTC in exon 8 in mRNA. However, we could identify mutant RT-PCR products in our patient, which means NMD does not always affect mutations with PTCs. On the other hand, it is possible that the pathogenic mechanism of PHA1 in patients with *MR *mutations depends on the mutation position. PHA1 is such a rare inherited disease that only about 40 mutations have been described by now, so that mRNA analytical studies of this disease are quite rare and additional studies are necessary to identify the mechanism of the phenotype of PHA1. It should be noted that the molar abundance of the smaller mRNA appears to be lower than the wild type transcript, that is possibly indicating that there is incomplete NMD of the mutant transcript in our study. Moreover, this study includes two limitations. First, we have not conducted quantitative PCR because we could not amplify clear PCR products with the one-step PCR, therefore, conducted nested PCR. Second, we have not determined the mutant peptide expression analysis because kidney tissues were unavailable in this disease. We must also hypothesize that, although not mediated by NMD, haploinsufficiency given to the loss of major functional domains of the receptor, may be the pathogenic mechanism of PHA1 in this patient. However, we can say, at least, some amount of mutant transcripts are expressed in the kidney tissues and the onset mechanism of haploinsufficiency because of complete NMD was denied in our patient.

By using mRNA extracted from urinary sediments, we could detect transcript abnormality noninvasively. Igarashi et al. were the first to identify transcript abnormalities by extracting mRNA from urinary sediment cells of patients with Dent disease [21]. Following their report, our group used this method to detect splicing abnormalities in mRNA from kidney cells [22-24]. Since urinary sediments contain cells derived from the kidney, genetic analysis of those cells constitutes an entirely noninvasive, simple method for the diagnosis of inherited kidney diseases, which can also be used for diagnosis of all other inherited kidney diseases including renal tubular disorders and glomerular diseases. Our study is the first to use this method for PHA1.

## Conclusion

Our report describes a novel *MR *mutation in a patient with PHA, and this mutation was found to eliminate a splice acceptor site, which results in exon 7 skipping and subsequently in premature termination in exon 8. This was demonstrated in mRNA from both peripheral blood leukocytes and urine sediments.

Our patient and her mother showed very mild symptoms in spite of a robust mutation of PHA1, which raises the possibility that *MR *mutation may not be involved in the severity of the symptoms of PHA1.

In this novel mutation, RT-PCR products of mRNA extracted from peripheral blood lymphocytes and urinary sediments showed evidence of the presence of wild-type and mutated abnormal mRNA. In our study we could provide clear evidence that NMD does not operate in mRNA. We could thus prove that NMD does not always observed in patients with *MR *mutations with PTCs. Additional studies including mRNA analysis will be needed to identify the exact mechanism of the phenotype of PHA.

## Abbreviations

PHA1: pseudohypoaldosteronism type 1; MR: human mineralcorticoid receptor gene; PRA: plasma renin activity; NMD: nonsense mediated mRNA decay; ACTH: adrenocorticotropic hormone; PCR: Polymerase chain reaction; PTC: premature termination codon.

## Competing interests

The authors declare that they have no competing interests.

## Authors' contributions

KK, KN, NY, IM, AM, YH, HK, KI and MM participated to the conduct and/or design of the study. NY, IM and AM are the clinical doctors of the patient. All authors have been involved in drafting the manuscript or revising it critically for important intellectual content. All authors have given final approval of the version to be published.

## Pre-publication history

The pre-publication history for this paper can be accessed here:

http://www.biomedcentral.com/1471-2369/10/37/prepub
